# Elevation of Proteasomal Substrate Levels Sensitizes Cells to Apoptosis Induced by Inhibition of Proteasomal Deubiquitinases

**DOI:** 10.1371/journal.pone.0108839

**Published:** 2014-10-06

**Authors:** Chao Sun, Peristera Roboti, Marjo-Riitta Puumalainen, Mårten Fryknäs, Xin Wang, Padraig D'Arcy, Malin Hult, Stephen High, Stig Linder, Eileithyia Swanton

**Affiliations:** 1 Cancer Center Karolinska, Department of Oncology and Pathology, Karolinska Institute, Stockholm, Sweden; 2 Faculty of Life Sciences, University of Manchester, Manchester, United Kingdom; 3 Department of Medical Sciences, Division of Clinical Pharmacology, Uppsala University, Uppsala, Sweden; 4 Center for Inherited Metabolic Diseases, Karolinska University Hospital, Stockholm, Sweden; Rajiv Gandhi Centre for Biotechnology, India

## Abstract

Inhibitors of the catalytic activity of the 20S proteasome are cytotoxic to tumor cells and are currently in clinical use for treatment of multiple myeloma, whilst the deubiquitinase activity associated with the 19S regulatory subunit of the proteasome is also a valid target for anti-cancer drugs. The mechanisms underlying the therapeutic efficacy of these drugs and their selective toxicity towards cancer cells are not known. Here, we show that increasing the cellular levels of proteasome substrates using an inhibitor of Sec61-mediated protein translocation significantly increases the extent of apoptosis that is induced by inhibition of proteasomal deubiquitinase activity in both cancer derived and non-transformed cell lines. Our results suggest that increased generation of misfolded proteasome substrates may contribute to the mechanism(s) underlying the increased sensitivity of tumor cells to inhibitors of the ubiquitin-proteasome system.

## Introduction

It has been estimated that as much as one-third of all proteins are destroyed within minutes of synthesis at the ribosomes [1]–[3]. These highly labile polypeptides include defective ribosomal translation products, as well as proteins that fold incorrectly during or shortly after synthesis. Misfolded proteins containing non-native structures are inherently cytotoxic [4], and quality control systems operate to identify and rapidly eliminate such aberrant proteins in order to maintain cellular homeostasis. Malignant transformation and tumor growth are associated with disregulated protein translation [5], which together with adverse intracellular conditions commonly experienced in the tumor environment, such as acidification [6] and increased levels of reactive oxygen species [7], may well result in increased generation of misfolded proteins. This hypothesis is further supported by the observation that tumor cells frequently exhibit signs of proteotoxic stress, including increased expression of Hsp70 and Hsp90 chaperones [8]–[10] and activation of the unfolded protein response (UPR). The level of proteotoxic stress in tumor cells may also be further exacerbated by aneuploidy and the resulting imbalance in components of protein complexes [11], [12].

The ubiquitin proteasome system (UPS) is the major intracellular protein degradation system responsible for the removal of defective and misfolded polypeptides in eukaryotes [13]. The 26S proteasome complex consists of a 20S core particle, which contains chymotrypsin-like, trypsin-like and peptidylglutamyl peptide hydrolysing activities [14], and two associated 19S regulatory particles, which control access to the proteolytic core. Proteins are targeted to the proteasome for degradation when they become modified with ubiquitin. Ubiquitin is a highly conserved 76 amino acid protein that is covalently attached to target proteins via a series of enzymatic steps, which culminate in the formation of an isopeptide bond between the C-terminus of ubiquitin and a lysine residue in the target protein [15]. Ubiquitin itself contains 7 lysine residues and additional ubiquitin monomers may be attached to any of these lysine residues, thus building up a polyubiquitin chain on the target protein. Chains of 4 or more ubiquitin molecules, typically linked through lysine 48 of ubiquitin, form highly specific signals for proteasomal degradation [16]. Subunits of the 19S particle act as ubiquitin receptors that bind these polyubiquitin chains and present the ubiquitinated proteasomal substrate to the 20S proteolytic core [16]. Ubiquitin is removed from substrate proteins prior to degradation by the action of deubiquinase (DUB) enzymes, which catalyse hydrolysis of the isopeptide bond and regenerate free ubiquitin monomers [15]. In humans, substrate deubiquitination is catalysed by three proteasome-associated DUBs, USP14 and UCHL5 (or UCH37), which are cysteine proteases, and a metalloprotease RPN11 (or POH1). The relationship between these proteasomal DUBs and their precise roles in regulating substrate degradation are complex and not yet fully understood [17].

Interfering with the UPS in cancer cells has been successfully exploited for therapeutic purposes. Bortezomib (Velcade) is a selective inhibitor of the 20S proteasome that shows cytotoxic activity against several malignant cell types and has been approved by the FDA for the treatment of patients with multiple myeloma [18]. A second protesome inhibitor, carfilzomib, was recently approved for relapsed multiple myeloma, and a number of additional agents are being developed. Despite their demonstrated therapeutic value, the mechanisms underlying the cytotoxicity of proteasome inhibitors are not well defined. A common view is that proteasome inhibition results in the stabilization of proteins that inhibit cell survival [18]–[21]. NF-*κ*-B is one such protein, and this transcription factor has received considerable attention with regard to its potential role in apoptosis induced by proteasome inhibitors [18]. Likewise, the involvement of Myc and Noxa in this process has been investigated [22], [23]. Another potential scenario is that the accumulation of aberrant proteasomal substrates mediates the cytotoxic effects of proteasome inhibitors, either as a consequence of their inherent toxicity, or via the activation of stress signalling pathways such as the UPR [24]. Yet another mechanism was recently proposed whereby a fatal depletion of amino acids, due to reduced recycling of amino acids through proteasomal protein degradation, underlies proteasome inhibitor-induced cell death [25].

We recently identified compound b-AP15, an inhibitor of the USP14 and UCHL5 cysteine deubiquitinases of the 19S proteasome [26]. Similar to inhibitors of the 20S proteasome, b-AP15 inhibits proteasome function in cells, leading to an accumulation of polyubiquitinated proteasome substrates, and the compound is effective in a number of solid tumor models and in multiple myeloma tumor models [19], [27]. One of the hallmarks of the DUB inhibitor b-AP15 is that it is more effective at inducing apoptosis of tumor cells than non-malignant cells [26], and here we have examined potential mechanisms underlying this selective cytotoxicity. We show that b-AP15 exposure induces greater accumulation of high molecular weight polyubiquitinated proteins in HCT116 colon carcinoma cells than in untransformed cells, suggesting that the cellular load of ubiquitinated proteasomal substrates may modulate the sensitivity of cells to b-AP15. To test this hypothesis, we used an inhibitor of protein translocation at the endoplasmic reticulum to increase the production of misfolded proteins, and found that this increased the sensitivity of cells to b-AP15. In contrast, cellular cysteine levels did not appear to be altered following exposure to b-AP15 and amino acid supplementation did not protect against b-AP15-induced cell death. Together, our results suggest that the polyubiquitinated proteasome substrates, which accumulate upon exposure of cells to b-AP15 contribute to the cytoxic effects of this drug.

## Results

### Elevated accumulation of ubiquitin conjugates in HCT116 tumor cells treated with an inhibitor of proteasomal deubiquitinases

**Figure 1:**
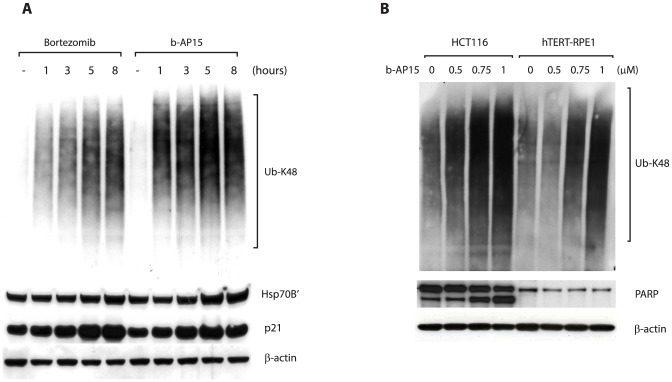
b-AP15 treatment causes accumulation of high molecular weight polyubiquitinated proteins and induces apoptosis. **A.** HCT116 cells were cultured in the presence of 1 µM b-AP15 or 100 nM bortezomib and harvested at the indicated time points. Lysates were subjected to immunoblotting for K48-linked polyubiquitin, HSP-70B', p21, PARP or β-actin (loading control). **B.** HCT116 cells or hTERT-RPE1 cells were exposed to different concentrations of b-AP15 for 1 hour, followed by washing and incubation for 16 hours in drug-free medium. Lysates were subjected to immunoblotting for K48-linked polyubiquitin, PARP and β-actin (loading control).

In order to gain insight into the mechanisms through which the USP14/UCHL5 inhibitor b-AP15 kills cells, we compared the effect of this compound on HCT116 colon cancer cells and non-malignant hTERT-RPE1 cells. Colon cancer cells are among the most sensitive to b-AP15 in the NCI_60_ panel (HCT116, HT29 and HCT15 cell lines show similar levels of sensitivity [26]), and hence were chosen for studies addressing the mechanism of b-AP15-induced apoptosis. Cells were treated with 1 µM b-AP15 for increasing periods of time, and lysates analysed by immunoblotting with antibodies that recognise K48-linked ubiquitin. A dramatic increase in cellular levels of high-molecular weight ubiquitin-conjugated proteins was observed within 1 hour of drug treatment (**Fig. 1A** **, right**). A similar time-course of accumulation of ubiquitin-conjugated proteins was observed when HCT116 cells were treated with bortezomib, a clinically used inhibitor of the 20S proteasome (**Fig. 1A** **, left**). The accumulation of these high molecular weight species required ongoing protein synthesis (not shown), suggesting that they include nascent polypeptides that were ubiquitinated during or soon after synthesis and were en route to proteasomal degradation. Both b-AP15 and bortezomib treatment induced the expression of Hsp70B', a stress-induced chaperone [28], and led to an increase in levels of the proteasome substrate p21 (**Fig. 1A** **; Fig. S1**). In contrast, Hsp90 expression was not obviously increased following treatment with b-AP15 (**Fig. S1**). These observations are consistent with the compounds blocking proteasomal degradation and thus increasing cellular levels of misfolded proteasome substrates. Continued exposure of HCT116 cells to b-AP15 resulted in apoptosis, as demonstrated by cleavage of poly(ADP-ribose) polymerase (PARP) (**Fig. 1B**). Thus, treatment of HCT116 colon cancer cells with b-AP15 leads to a rapid accumulation of poly-ubiquitinated proteins, upregulation of Hsp70B', and ultimately induction of programmed cell death. We previously demonstrated that untransformed cells are less sensitive to b-AP15-induced apoptosis than cancer cells such as HCT116 cells [26]. Consistent with this, cleavage of PARP was not detected in immortalized epithelial hTERT-RPE1 cells following exposure to b-AP15, despite being clearly apparent in HCT116 cells treated under the same conditions (**Fig. 1B**).

Interestingly, the accumulation of polyubiquitinated proteins induced by b-AP15 was also lower in hTERT-RPE1 cells than in HCT116 cells treated in parallel (**Fig. 1B**). Thus, the increased sensitivity of HCT116 cells to b-AP15-induced cell death correlated with greater accumulation of polyubiquitin conjugates (**Fig. 1B**). Cancer cells are characterised by disregulated translational control and increased rates of protein synthesis, and thus the production of polypeptides that require proteasomal degradation is also likely to be greater in these cells [5]. On this basis, we speculated that it might be the load of proteasome substrates generated by HCT116 cells that determines their sensitivity to b-AP15, a hypothesis that is supported by the correlation between the accumulation of polyubiquitin-conjugated proteins and PARP cleavage following exposure to b-AP15 (**Fig. 1B**).

### An inhibitor of protein translocation at the endoplasmic reticulum increases the accumulation of ubiquitin conjugates in cells treated with UPS inhibitors

**Figure 2:**
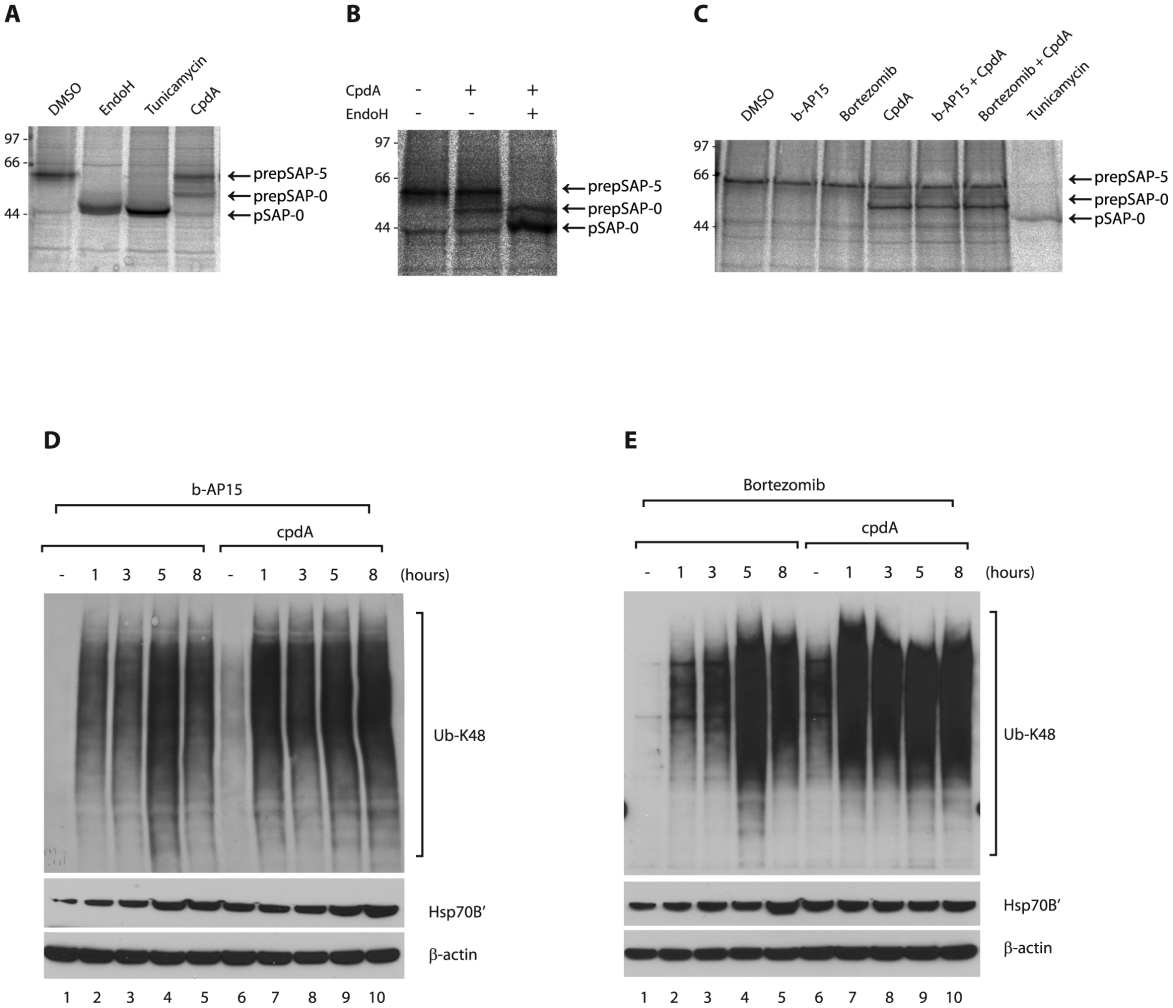
CpdA inhibits co-translational translocation of endogenous prosaposin into the ER. **A. and B.** HCT116 cells were treated for 1 h with DMSO, tunicamycin (10 µg/ml) or CAM741 (10 µM) before pulse-labelling for 10 min with [^35^S] Met/Cys. Endogenous prosaposin (pSAP) was recovered by immunoprecipitation and newly synthesised pSAP species were visualised by phosphorimaging. In DMSO-treated cells, pSAP was fully glycosylated (pSAP-5), whereas Endo H digestion or tunicamycin treatment yielded non-glycosylated pSAP (pSAP-0). Inhibition of protein translocation into the ER by CAM741 resulted in the appearance of a pSAP species that migrated more slowly than the non-glycosylated protein and was Endo H-resistant. This species may represent signal sequence-containing preprosaposin (prepSAP-0) that has failed to translocate across the ER membrane. **C.** Distinct forms of endogenous pSAP in HCT116 cells treated with DMSO, b-AP15 (1 µM), bortezomib (20 nM), cpdA (10 µM) or tunicamycin (10 µg/ml) were recovered by immunoprecipitation and visualised by phosphorimaging. Treatment with cpdA specifically inhibits the co-translational translocation of pSAP into the ER as judged by the appearance of prepSAP-0 species.

In order to address the role of proteasomal substrate levels more directly, we examined the effect of increasing the production of putative substrates using cpdA, an inhibitor of Sec61-mediated protein translocation at the endoplasmic reticulum (ER) [29]. The effect of cpdA on ER translocation in HCT116 cells was determined by monitoring the fate of an endogenous glycoprotein, prosaposin. In the absence of cpdA, the prosaposin precursor (preprosaposin, prepSAP) is efficiently translocated into the ER lumen, the signal peptide removed and the protein becomes N-glycosylated at five sites, generating prosaposin (**Fig. 2A** **, DMSO, labelled pSAP-5**). Treatment with Endoglycosidase H or inhibition of N-glycosylation with tunicamycin produced a un-glycosylated form of prosaposin (**Fig. 2A** **, labelled pSAP-0**). Inhibition of ER translocation by cpdA has previously been shown to result in the mislocalisation of precursor proteins to the cytosol, where they are targeted for proteasomal degradation [30]. In line with these observations, treatment with cpdA led to the appearance of an additional form of prosaposin in HCT116 cells (**Fig. 2A** **, labelled prepSAP-0**). The additional form generated in the presence of cpdA was not sensitive to cleavage by Endoglycosidase H (**Fig. 2B**), showing that it lacks nascent N-glycans. Furthermore, this species migrates more slowly than the un-glycosylated prosaposin (**Fig. 2B** **, compare pSAP-0 and prepSAP-0**), suggesting that it still retains its signal peptide. Thus, we conclude that this additional form represents preprosaposin that has failed to be translocated across the ER membrane, and is therefore unable to undergo post-translational modifications within the ER lumen. These results suggest that cpdA inhibits the translocation of endogenous proteins across the ER membrane of HCT116 cells, leading to the generation of mislocalised polypeptides. The steady state levels of non-translocated preprosaposin were not obviously increased by exposure to b-AP15 or bortezomib (**Fig. 2C**), consistent with its rapid polyubiquitination [30] to generate a range of higher molecular weight species that are not readily visible. Indeed, HCT116 cells pre-treated with 10 µM cpdA for 16 h appeared to contain slightly increased levels of polyubiquitin conjugates than untreated cells (**Fig. 2D, 2E** **, compare lanes 1 and 6**). Taken together, these data suggest that the treatment of HCT116 cells with cpdA leads to increased generation of proteasome substrates.

We next examined the effect of b-AP15 treatment on HCT116 cells in the presence of cpdA to increase the load of endogenous proteasomal substrates. In the presence of cpdA, b-AP15 treatment leads to an even greater increase in the levels of polyubiquitinated proteins, as compared to cells treated with b-AP15 alone (**Fig. 2D**). A similar effect of cpdA to promote the accumulation of polyubiquitin conjugates was also observed upon treatment with bortezomib (**Fig. 2E**). This increase in the level of polyubiquitinated components upon co-exposure to cpdA was particularly evident 1 h after addition of b-AP15 or bortezomib (**Fig. 2D, 2E** **, compare lanes 2 and 7**).

**Figure 3:**
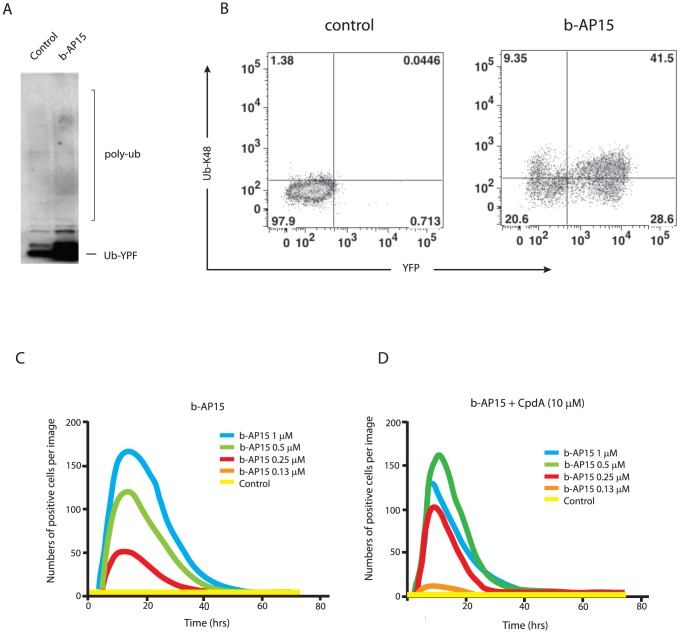
CpdA enhances the ability of b-AP15 to inhibit proteasomal degradation. **A.** MelJuSo cells stably expressing Ub^G76V^-YFP were treated with 1 µM b-AP15 for 6 hours or left untreated (control), and cell lysates subjected to immunoblotting using anti-YFP. The migration of Ub^G76V^-YFP and the expected position of higher molecular weight polyubiquitinated forms (poly-Ub) are indicated. **B.** MelJuSo-Ub^G76V^-YFP cells were exposed to 1 µM b-AP15 for 8 hours or left untreated (control). Cells were labeled with an anti-K48 polyubiquitin antibody followed by an allophycocyanin conjugated secondary antibody and analyzed by FACS. **C. and D.** MelJuSo-Ub^G76V^-YFP cells were exposed to different concentrations of b-AP15 in the presence or absence of 10 µM cpdA as indicated. Changes in the number of fluorescence-positive cells/field following addition of the compounds were monitored using an IncuCyte-FLR microscope.

In order to examine the effect of cpdA on the accumulation of proteasomal substrates more directly, we utilized Ub^G76V^-YFP. This model fusion protein is constitutively degraded by the proteasome, and hence steady state levels of Ub^G76V^-YFP can be used to quantify ubiquitin proteasome-dependent proteolysis in live cells [31]. Treatment of MelJuSo cells stably expressing Ub^G76V^-YFP with b-AP15 for 6 hours resulted in a marked accumulation of the reporter (**Fig. 3A**), confirming effective inhibition of proteasomal degradation. Although most of the Ub^G76V^-YFP detected by immunoblotting was not polyubiquitinated (**Fig. 3A**), most, but not all, cells that became Ub^G76V^-YFP positive after exposure to b-AP15 also stained with an antibody to lysine 48-linked polyubiquitin chains (**Fig. 3B**). The effect of b-AP15 on the accumulation of Ub^G76V^-YFP was determined in real-time using an IncuCyte instrument to quantify the number of YFP-positive cells (**Fig. 3C**). Accumulation of Ub^G76V^-YFP occurred rapidly following addition of the drug, and YFP-positive cells could be detected in cells treated with 0.25 µM b-AP15 (**Fig. 3C** **, red trace**). The number of YFP positive cells increased up to 1 µM b-AP15 (**Fig. 3C** **, blue trace**), and then Ub^G76V^-YFP accumulation decreased at higher concentrations of b-AP15, potentially due to its toxicity at these levels. Interestingly, when cells were co-treated with cpdA, YFP-positive cells were now detected following treatment with only 0.13 µM b-AP15 (**Fig. 3D** **, orange trace**). Hence, in the presence of cpdA, the threshold concentration of b-AP15 required for detection of Ub^G76V^-YFP accumulation was decreased by approximately one half (**Fig. 3C** **compared with** **Fig. 3D**). In contrast, treatment with 10 µM cpdA alone did not induce accumulation of Ub^G76V^-YFP (**Fig. 3C, D** **, compare yellow traces**). We therefore conclude that cpdA increases the accumulation of proteasomal substrates, such as Ub^G76V^-YFP, induced by b-AP15. Together these results suggest a mechanism whereby cpdA enhances the ability of b-AP15 to inhibit proteasomal degradation by increasing the burden of proteasome substrates, which may potentially saturate or block the proteasome. Hence, cpdA may promote accumulation of polyubiquitinated proteins both by increasing the production of mislocalised proteasome substrates and also by reducing proteasomal degradation.

**Figure 4:**
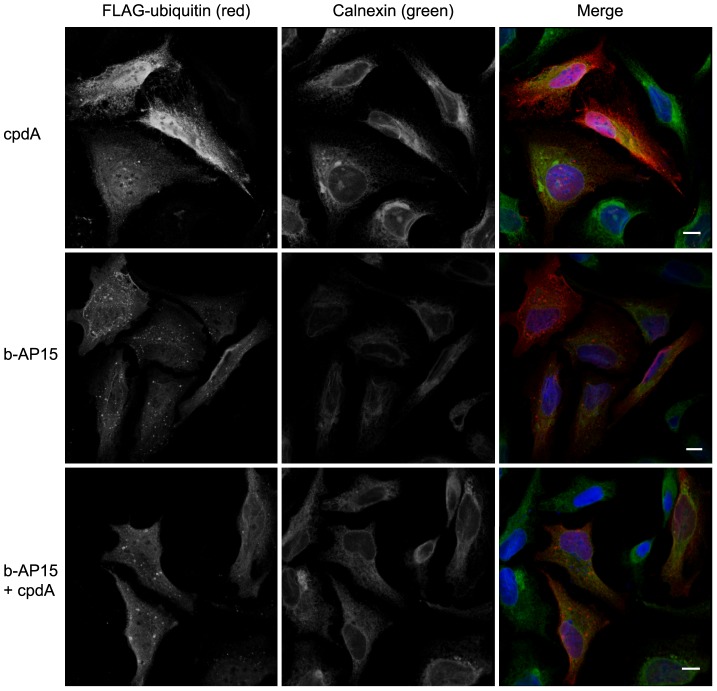
b-AP15 treatment alters the subcellular distribution of ubiquitin and causes the appearance of ubiquitin positive inclusions. HeLa-M cells transiently transfected with FLAG-ubiquitin were treated with 10 µM cpdA (top panels), 1.0 µM b-AP15 (middle panels) or 0.8 µM b-AP15 plus 10 µM cpdA (bottom panels) for 16 h. Cells were fixed and stained with anti-FLAG and anti-calnexin antibodies followed by fluorescently labelled secondary antibodies. Confocal images were collected and images show the combined optical stacks. Scale bar  = 10 µM.

To gain further information about the nature of the polyubiquitinated proteins that accumulated in the presence of b-AP15 and cpdA, we examined the subcellular distribution of ubiquitin using indirect immunofluorescence microscopy. HeLa cells transiently expressing FLAG-tagged ubiquitin were treated with compounds for 6 h, fixed and stained with anti-FLAG to visualise ubiquitin and anti-calnexin as a subcellular marker for the ER. The distribution of FLAG-ubiquitin in cells treated with 10 µM cpdA for 6 hours resembled that of untreated cells (**Fig 4** **; not shown**). Hence, FLAG-ubiquitin was enriched in the nucleus and was also apparent throughout the cytoplasm (**Fig. 4** **, top row**). In contrast, treatment with 1 µM b-AP15 resulted in the appearance of numerous ubiquitin-positive foci. These punctate structures were of variable size, and were distributed throughout the cytoplasm and in the perinuclear region (**Fig. 4** **, middle row**). FLAG-ubiquitin containing foci were also evident in cells co-treated with a combination of b-AP15 and cpdA (**Fig. 4** **, bottom row**). However, their appearance was qualitatively different, with fewer larger FLAG-ubiquitin containing punctae being observed in the presence of cpdA. The formation of similar ubiquitin-positive foci has been observed in response to a variety of conditions that disrupt protein folding and/or degradation [32]. In many cases, these structures contain misfolded proteins. Thus, the ubiquitin-positive puncta observed here may represent deposition sites for the polyubiquitinated proteins that accumulate following treatment with b-AP15. The subtle difference in the appearance of the punctae formed in the presence of cpdA may reflect the accumulation of a distinct type of ubiquitinated proteasome substrates, including mislocalised ER targeted proteins such as preprosaposin.

### Inhibition of protein translocation with cpdA sensitizes cells to b-AP15-induced apoptosis

**Figure 5:**
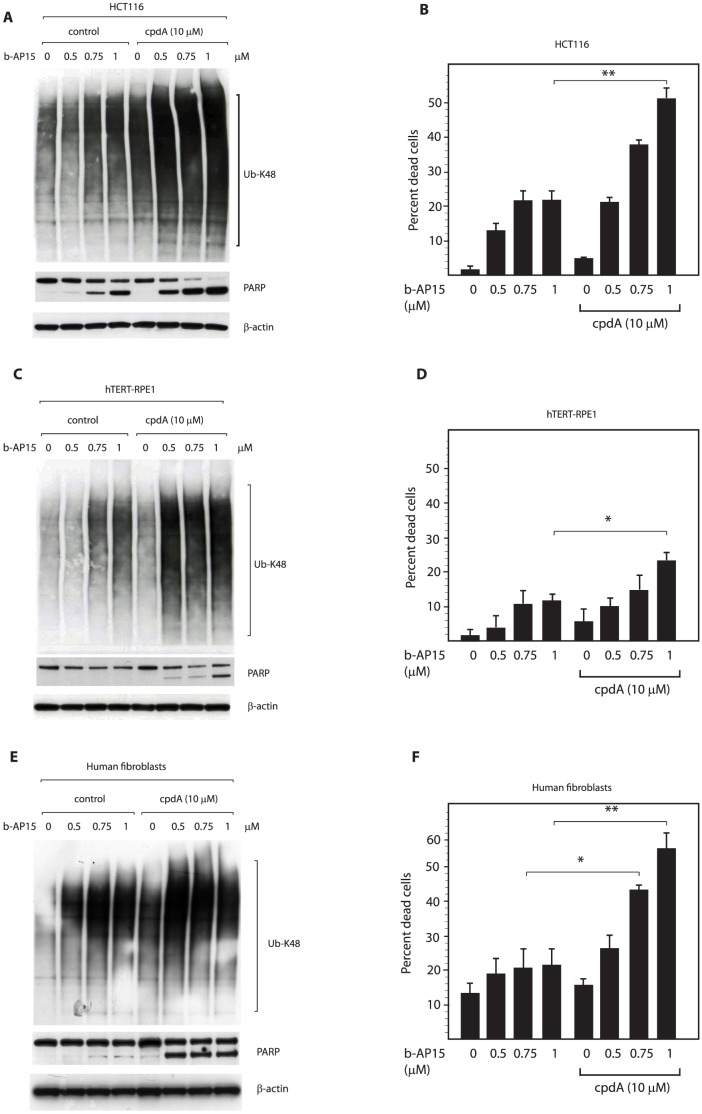
CpdA increases cellular levels of polyubiquitinated proteins and sensitizes cells to b-AP15 induced cell death. **A.** HCT116 cells were pre-treated with or without (control) 10 µM cpdA for 16 hours, exposed to the indicated concentration of b-AP15 for 1 hour, then incubated for a further 16 hours in drug-free medium. Lysates were subjected to immunoblotting for K48-linked polyubiquitin, PARP and β-actin (loading control). **B.** HCT116 cells were treated as above. Following treatment, the number of dead cells was measured by Trypan-blue staining. Bar chart shows mean +/− SD of three independent experiments. Statistical significance was calculated using the Student's t-test. P values * = 0.05 and ** = 0.01. **C. and D.** hTERT-RPE1 cells were treated and analyzed as above. **E. and F.** Human diploid fibroblasts were treated and analyzed as above, and the number of dead cells was determined 36 hours after drug treatment.

In order to examine the consequences of increased accumulation of polyubiquitinated conjugates and decreased proteasomal degradation, we next measured the effect of cpdA treatment on b-AP15-induced cell death. In line with previous observations [26], exposure of HCT116 cells to b-AP15 rapidly (within 16 hours) induced cleavage of PARP (**Fig. 5A**) and cell death (**Fig. 5B**) in a dose-dependent manner. Interestingly, pre-treatment of cells with cpdA prior to exposure to b-AP15 significantly increased the extent of PARP cleavage (**Fig. 5A**) and cell death (**Fig. 5B**) induced by b-AP15. Hence, in the absence of cpdA, 0.75 µM b-AP15 was required to effectively stimulate PARP cleavage, whilst 0.5 µM was sufficient in cells pretreated with cpdA (**Fig. 5A**). More strikingly, cpdA pre-treatment greatly sensitized HCT116 cells to b-AP15 induced cell death, with the percentage of dead cells increasing from approximately 20% after exposure to 0.75–1.0 µM b-AP15 to 40–50% in the presence of cpdA (**Fig. 5B**). A direct correlation between the accumulation of polyubiquitinated proteins and the extent of cell death during these treatment regimens was observed, with cpdA enhancing the build-up of lysine 48-linked polyubiquitin conjugates (**Fig. 5A, B**). This correlation also extended to non-transformed hTERT-RPE1 cells, in which lower levels of polyubiquitin conjugates accumulated and b-AP15 failed to induce PARP cleavage or evident cell death in the absence of cpdA even after 24 hours (**Fig. 5C, D** **; Fig. S2**). However, when hTERT-RPE1 cells were exposed to cpdA the levels of polyubiquitin conjugates induced by b-AP15 were increased, and both PARP cleavage and cell death were now observed (**Fig. 5C, D**). Primary human fibroblasts were even more resistant than RPE1 cells to b-AP15-induced cell death (**Fig. S2**). However, 36 h after drug treatment, a similar synergistic toxicity of cpdA and b-AP15 was apparent (**Fig. 5E, F**), showing that both untransformed cell types can be sensitized to b-AP15-induced apoptosis by treatment with cpdA. Taken together, these data are consistent with the hypothesis that the amount of proteasome substrates that are generated in cells modulates sensitivity to b-AP15.

### b-AP15 treatment does not deplete the intracellular cysteine pool in HCT116 cells

**Figure 6:**
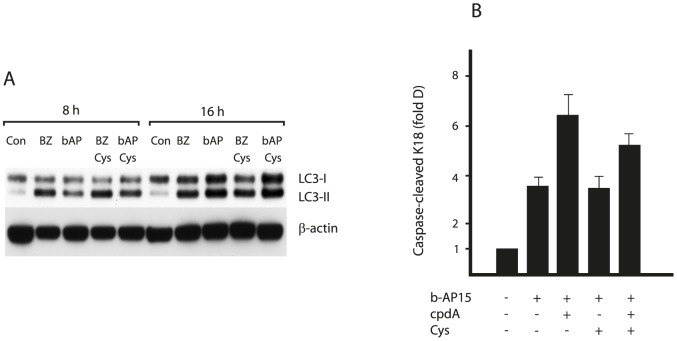
Cysteine supplementation does not protect HCT116 cells from b-AP15 toxicity. **A.** HCT116 cells were exposed to 1 µM b-AP15 or 100 nM bortezomib (BZ) for 8 hours or 16 hours, in the presence or absence of 1 mM cysteine as indicated. Cell lysates were subjected to immunoblotting for LC3-I/II and β-actin (loading control). **B.** HCT116 cells were exposed to 1 µM b-AP15 for 16 hours in the presence or absence of 1 mM cysteine. Where indicated, cells were pre-treated with 10 µM cpdA for 16 hours prior to b-AP15 treatment. Apoptosis was determined by the appearance of caspase-cleaved K18. Bar chart shows mean values ± SD of of three independent experiments.

The data presented above support a model whereby the cytotoxicity of b-AP15 is due to an inhibition of proteasome function and the resulting accumulation of polyubiquitinated proteins. These species will include misfolded and short lived proteins en route to proteasomal degradation, and may be directly toxic to cells. Alternatively, reduced proteasomal degradation following exposure to b-AP15 may result in a lethal depletion of amino acids, most notably cysteine, due to the deficient recycling of amino acids from UPS substrates, as has been found to occur when proteasome activity is inhibited directly [25]. Indeed, we found that b-AP15 treatment resulted in an increase in LC3-II levels, indicative of autophagy, as might be expected under conditions of amino acid shortage (**Fig. 6A**). In order to address this issue more directly, we therefore examined the effects of b-AP15 and cpdA on the cellular pools of cysteine (**Table 1**). However, no decrease in cysteine levels could be detected following exposure of HCT116 cells to b-AP15 for 8 h, either alone or in combination with cpdA. Furthermore, supplementing cultures with 1 mM cysteine and other essential amino acids did not protect HCT116 cells from b-AP15-induced cell death (**Fig. 6B**), or reduce the induction of autophagy observed after b-AP15 treatment (**Fig. 6A**). Similar results were obtained using bortezomib (**Fig. 6A, B**), and we found that cellular cysteine levels were actually higher in HCT116 cells treated with bortezomib (**Table 1**). The reasons for the apparent discrepancy with recent work [25] are not clear, but may well be due to differences in cell type (i.e. human tumor versus mouse NIH3T3 cells), the precise experimental conditions used, or the stage in proteasomal degradation that is inhibited by b-AP15. Nonetheless, these results strongly suggest that cysteine depletion is not the sole cause of apoptosis in HCT116 cells exposed to b-AP15.

**Table 1:** Treatment of HCT116 cells with b-AP15 and cpdA does not deplete cellular cysteine levels.

Sample	Control	b-AP15	BZ	b-AP15 + cpdA	BZ + cpdA
Cysteine (µmol/g)	0.46±0.11	0.65±0.17	1.35±0.48	0.54±0.18	0.53±0.22

HCT116 cells were treated with 1 µM b-AP15 or 100 nM bortezomib (BZ) for 8 hours Where indicated, cells were pre-treated with 10 µM cpdA for 16 hours prior to b-AP15 or bortezomib treatment. Cells were collected and intracellular cysteine levels measured as described. Data are mean ± SD of three independent experiments.

## Discussion

The UPS is a major pathway for protein degradation in eukaryotic cells. Tumor cells depend heavily on UPS function, and inhibitors of the proteolytic activity of the proteasome have demonstrated efficacy for the treatment of certain types of cancer. We recently identified b-AP15, a novel type of UPS inhibitor that targets DUB enzymes of the 19S regulatory particle of the proteasome, but does not inhibit the activity of the 20S core particle [26]. b-AP15 displays anti-cancer activity in several models of cancer including solid-tumors, suggesting that inhibition of 19S DUB activity may provide an alternative therapeutic strategy to proteasome inhibitors [26]. Treatment of cancer cells with b-AP15 has been shown to inhibit degradation of UPS substrates and to induce accumulation of very high molecular weight ubiquitin-conjugated proteins [26]. Thus, it was suggested that a build-up of proteasome substrates may underlie the cytotoxicity of b-AP15 [17], [26]. Here we have addressed this hypothesis and provide evidence that sensitivity to b-AP15-induced cell death is related to the accumulation of high molecular weight polyubiquitinated proteins induced by this inhibitor of proteasomal DUBs.

Previous work demonstrated that b-AP15 preferentially kills cancer cells [26], and we confirm here that non-transformed hTERT-RPE1 cells and primary human fibroblasts are less sensitive to b-AP15-induced apoptosis than HCT116 colon carcinoma cells. Furthermore, we identify a correlation between the sensitivity of cells to b-AP15-induced cell death and the accumulation of high molecular weight ubiquitin conjugates, consistent with the suggestion that a harmful build-up of ubiquitinated proteasome substrates contributes to the cytotoxic effects of b-AP15. Hence, in contrast to HCT116 cells, non-malignant cells accumulated much lower levels of polyubiquitinated proteins upon exposure to b-AP15 and did not undergo extensive apoptosis following drug treatment.

We provide further evidence to support this hypothesis using an inhibitor of protein translocation at the ER to show that increasing the cellular load of proteasome substrates modulates sensitivity to b-AP15. Up to a third of cellular proteins are translocated into or across the ER membrane during their biosynthesis, and those that fail to translocate correctly are ubiquitinated and targeted for proteasomal degradation [30]. Thus, we reasoned that inhibition of ER translocation would promote the production of UPS substrates and increase the accumulation of polyubiquitinated proteins in cells treated with b-AP15. The small molecule cpdA was originally identified as an inhibitor of vascular cell adhesion molecule 1 (VCAM1) translocation [29], [33], and at low micromolar concentrations causes a more general inhibition of ER protein translocation [34], [35]. Consistent with an inhibition of cotranslational translocation in HCT116, treatment with cpdA caused production of a mislocalised form of the endogenous glycoprotein saposin. Furthermore, pre-treatment with cpdA exacerbated the build-up of polyubiquitinated proteins induced by bortezomib or by b-AP15, suggesting that the cellular load of proteasome substrates was indeed increased under these conditions. Strikingly, cpdA also significantly sensitized cells to b-AP15 induced cell death, suggesting a scenario whereby the accumulation of polyubiquitinated proteasome substrates contributes to the toxicity of b-AP15.

Although the precise identity of the polyubiquitinated proteins that accumulate in response to b-AP15 treatment is unknown, we speculate that these will include misfolded and other non-native proteins en route to proteasomal degradation. Such species often possess exposed hydrophobic regions and thus have a tendency to form aggregates. Indeed, we observed the appearance of ubiquitin-positive inclusions within cells following b-AP15 treatment. The formation of similar structures has been observed in the presence of 20S inhibitors and under conditions that disrupt protein folding [36], and we suggest that they represent deposition sites for the polyubiquitinated proteins that accumulate in the presence of b-AP15. It is well established that the accumulation of misfolded proteins and protein aggregation is potentially harmful and can impair cell function, a process termed proteotoxicity or proteotoxic stress. Proteotoxicity of misfolded proteins has been best documented in relation to neurodegenerative diseases, and numerous mechanisms have been proposed to underlie cellular dysfunction in these situations [37]. Prominent among these is the propensity of non-native proteins to engage in inappropriate interactions and thus sequester key cellular factors and/or interfere with essential cellular processes [38], [39]. In addition, studies have shown that protein aggregates can disrupt membrane integrity, induce mitochondrial dysfunction and increase production of reactive oxygen species [40]–[45]. Which, if any, of these mechanisms contribute to b-AP15-induced cell death will be the focus of our future studies.

An alternative possibility is that b-AP15 exerts its anti-tumor activity by inhibition of anti-apoptotic proteins such as NF-κB, by stabilization of tumor suppressors such as p53, or by stabilization of Myc and Noxa, as has been suggested for the killing of cancer cells by 20S proteasome inhibitors [18]–[23]. Whilst it is conceivable that these mechanisms contribute to b-AP15-induced cell death, they do not readily explain how cpdA increases b-AP15 toxicity. Hence, we favour a model whereby proteotoxicity of undegraded proteasome substrates underlies the ability of b-AP15 to kill cancer cells. In tumor cells, increased translational flux and adverse intra- and extracellular conditions may lead to an increased production of misfolded proteins, and in these situations inhibition of protesomal degradation would be predicted to cause greater toxicity. Our interpretation that cpdA potentiates b-AP15 cytoxicity by increasing cellular levels of misfolded proteins is consistent with previous work showing that inducing protein misfolding, for example by downregulation of molecular chaperones [28], [46] or hyperthermia [8], enhances the severity of proteasome inhibitor-induced proteotoxic stress and cell death.

Depletion of essential amino acids due to inefficient recycling has recently been shown to play a key role in proteasome inhibitor mediated cell death in yeast, fruit flies and mouse cells [25]. However, a lack of amino acids does not appear to be the major underlying cause of b-AP15-induced apoptosis in the HCT116 colon cancer cells studied here. In this respect it is noteworthy that in human cancer cells, the outcome of treatment with proteasome inhibitors appears to depend on the balance between the accumulation of misfolded proteins and the protective upregulation of chaperones [28], [46]. We observed strong induction of Hsp70B' in HCT116 cells treated with b-AP15. Hsp70B' is highly inducible and considered as the final defence line against proteotoxic stress in human cells, whereas the mouse genome does not encode Hsp70B'. Hence, such cells may respond differently to the accumulation of proteasome substrates as compared to the human cells we have employed here.

b-AP15 kills cancer cells by inducing apoptosis [26], [27], [47]. Our recent work shows that the intrinsic pathway is engaged, with conformational activation of Bak and mitochondrial depolarisation evident upon b-AP15 treatment [48]. Interestingly, the toxic activity of b-AP15 is distinct from that of 20S core particle inhibitors. In contrast to bortezomib, b-AP15 induces cell death independently of Bcl-2, Bax and Bak, exhibits anti-tumor activity in solid tumor models and shows greatest activity towards colon carcinoma and CNS lineage cells [17], [48]. Although the reasons for these differences are not known, one intriguing possibility is that specific properties of the ubiquitin conjugates that accumulate in the presence of b-AP15 contribute to its selective toxicity. Indeed, the polyubiquitinated proteins observed in b-AP15 treated cells are typically of higher molecular weight than those that accumulate as a result of inhibition of the 20S core particle [26]. It is possible that such extensively polyubiquitinated species have distinct effect on cell function compared to lower order ubiquitin conjugates. In addition, cytoplasmic aggregates containing misfolded proteins have been found to compromise cellular function by sequestering essential cellular components including molecular chaperones [38], [39], [41]. Hence, it will be important to determine the identity of other cellular factors that are present in the ubiquitin positive inclusions formed upon b-AP15 treatment.

Similarly, it is possible that the nature of the proteasome substrates generated in the presence of cpdA contribute to its ability to potentiate b-AP15 cytotoxicity. Mislocalised polypeptides may have an increased risk of aggregation in the cytosol due to the presence of hydrophobic transmembrane domains and/or unprocessed signal peptides, and thus may induce greater proteotoxicity. Indeed, a dedicated quality control system for proteins possessing hydrophobic ‘degrons’ has recently been identified [30], [49], underscoring the requirement for timely and efficient elimination of such species. In this respect, it is noteworthy that we observed a subtle difference in the subcellular localisation of ubiquitin in cells treated with b-AP15 and cpdA, with larger ubiquitin positive inclusions detected than with b-AP15 alone. Furthermore, co-treatment with cpdA slightly enhanced the ability of b-AP15 to inhibit degradation of the UPS reporter substrate Ub^G76V^-YFP. These observations are interesting as they raise the possibility that the mislocalised proteins generated in the presence of cpdA may interfere with proteasome function and/or sequester other factors required for proteasomal degradation as recently demonstrated for other protein aggregates [39], [41].

In principal, enhancement of b-AP15-induced apoptosis by cpdA co-treatment could provide a potential strategy for improved cancer therapy. Supporting such a combinatorial approach, induction of protein misfolding by genetic or pharmacological inhibition of molecular chaperones has been shown to enhance the anti-tumor effect of bortezomib [50]. Increasing the levels of proteasome substrates in cells by inhibiting protein translocation may thus represent a novel strategy to increase proteotoxic stress and sensitize cells to bortezomib-induced apoptosis. However, we found that cpdA also increased bortezomib toxicity in two non-malignant cell types, and it is therefore unclear whether this combination of drugs could be clinically useful in practice. Rigorous testing of this possibilty will require animal experiments and clinical studies.

## Experimental Procedures

### Antibodies and plasmids

Monoclonal anti-FLAG (M2), anti-HSPA6, anti-β-actin and polyclonal anti-calnexin antibodies were purchased from Sigma Aldrich. Goat anti-saposin antibody was from Konrad Sandhoff (University of Bonn, Germany). The K48-linked polyubiquitin antibody (clone Apu2) was from Millipore. Antibodies for LC3 and GFP were from Cell Signalling Technology. Anti-PARP, anti-Caspase3 and anti-HSP90 were from Becton Dickinson. The anti-HSP70B' was from Lifespan. The plasmid encoding FLAG-tagged ubiquitin was kindly provided by Sylvie Urbe (University of Liverpool).

### Cell culture

HCT116 colon carcinoma cells (ATCC) were maintained in McCoy's 5A modified medium/10% fetal calf serum, hTERT-RPE1 cells [26] in DMEM:F12 medium/10% fetal calf serum, human diploid fibroblasts and HeLa cells (European Collection of Cell Cultures) were maintained in DMEM medium/10% fetal calf serum and MelJuSo-Ub^G76V^-YFP cells [51] in Iscove's DMEM medium/10% fetal calf serum.

### Treatment with small molecules

b-AP15 (NSC687852) was obtained from the Developmental Therapeutics Program of the US National Cancer Institute (http://www.dtp.nci.nih.gov/) or from OncoTargeting AB, bortezomib was from the Department of Oncology, Karolinska Hospital, and cpdA was kindly provided by Novartis. Stock solutions of all compounds were made in DMSO and were added directly to culture media at the concentrations indicated.

### SDS-PAGE and Western blot analysis

Following treatment, cells were lysed in RIPA buffer (140 mM NaCl, 10 mM Tris-Cl pH 8.0, 1 mM EDTA, 0.5 mM EGTA, 1% Triton X-100, 0.1% sodium deoxycholate, 0.1% SDS) containing protease inhibitor cocktail (Sigma). Lysates were resolved on Tris-Acetate PAGE gels (Invitrogen, Carlsbad, CA) and transferred onto a polyvinylidene difluoride (PVDF) membrane for Western blotting.

### Assessment of apoptosis by ELISA

HCT116 cells were seeded in 96-well microtiter plates at 10,000 cells per well and incubated overnight. Drugs were then added and cells incubated further. At the end of the incubation period, NP40 was added to the tissue culture medium to 0.1% and 25 µl of the content of each well was assayed using the M30-Apoptosense ELISA [52]. This ELISA is based on a specific antibody against a neoepitope of cytokeratin 18 that is generated by the action of caspase-3, -7 and -9 activated in response to apoptosis [53].

### FACS

MelJuSo-Ub^G76V^-YFP cells treated with or without b-AP15 were collected and fixed in 2% paraformaldehyde for 15 min at room temperature. After permeabilization with 0.2% Triton X-100/2% BSA in PBS, cells were stained with an anti-K48 antibody (Milipore, 1∶700) for 1 hour at room temperature in PBS/2% BSA, washed 3 times with PBS and incubated with an anti-rabbit antibody conjugated to allophycocyanin (Thermo Scientific, 1∶200) for 30 min in PBS/2% BSA at room temperature. Flow cytometry was performed using an BD LSR II instrument.

### Immunofluorescence microscopy

For immunofluorescence microscopy, HeLa M cells were grown on coverslips and transfected with jetPEI (Polyplus Transfection) according to the manufacturer's instructions. Eight hours post-transfection, cells were treated with compounds for 16 h. Cells were fixed in methanol at −20°C for 4 min, and then incubated for 1h at room temperature with primary antibodies diluted in PBS. Cells were washed three times with PBS and incubated for a further hour at room temperature with secondary antibodies conjugated to Alexa Fluor 488/594 (Invitrogen). The DNA dye DAPI (1 µg/ml) was included in the second incubation. Coverslips were mounted with Mowiol, allowed to dry, and images were acquired on a Nikon C1 confocal on an upright 90i microscope with a Nikon Apo oil 60x/1.40NA objective and the following settings: pinhole 30 µm, scan speed 1.68 s unidirectional, format 512×512. Images for DAPI, FITC and Texas Red were excited with the 405 nm, 488 nm and 543 nm laser lines, respectively. Image analysis was performed using Adobe Photoshop CS4.

### Quantification of Ub^G76V^-YFP fluorescence

Fluorescence of MelJuSo-Ub^G76V^-YFP cells was recorded as positive cells/field using an IncuCyte-FLR 20X phase contrast/fluorescence microscope (Essen Instruments, Ann Arbor, MI). Average object summed intensity was calculated (triplicate wells, 4 images/well) using the Incucyte software (Essen Instruments).

### Metabolic labelling and immunoprecipitation

Cells were starved in methionine- and cysteine-free DMEM (Invitrogen) supplemented with 2 mM L-glutamine for 20 min at 37°C, and then incubated in fresh starvation medium containing 22 µCi/ml [^35^S]Met/Cys protein labelling mix (PerkinElmer; specific activity >1,000 Ci/mmol) for 10 min. When the effects of different compounds were studied, the cells were pretreated for 1 h prior to starvation, and the compound(s) were included throughout the starvation and pulse. Cells were washed twice with PBS and solubilised with Triton X-100 lysis buffer containing 10 mM Tris-HCl pH 7.6, 140 mM NaCl, 1 mM EDTA, 1% Triton X-100, and a protease inhibitor cocktail. Clarified lysates were denatured with 1% SDS at 37°C for 30 min, then diluted 5-fold with Triton X-100 lysis buffer containing 8 mM cold Met/Cys and 1 mM PMSF, and pre-cleared by incubation with pansorbin (Calbiochem) at 4°C for 1 h. The pre-cleared lysates were incubated overnight with antibodies specific for saposin D and immune complexes were captured using Protein A-Sepharose beads (GenScript, USA). Beads were washed three times with Triton X-100 buffer before eluting proteins with SDS-PAGE sample buffer. The immunoprecipitated material was denatured at 37°C for 1 h, and then analysed directly or digested with 0.5 µl of Endo H and incubated at 37°C overnight. Samples were resolved by SDS-PAGE, and visualised by phosphorimaging (FLA-3000; Fuji). Phosphorimaging exposures were processed using AIDA v3.52.

### Measurement of intracellular cystein levels

The method for measurement of non-protein bound total cystine (cysteine and cystine) was developed from that of Araki and Sako [54]. HCT116 cells were washed in PBS and resuspended in 800 µl ice-cold water, then immediately disrupted by ultrasonication on ice/water slush (Soniprep 150 MSE, three times for 3 sec, amplitude 10 microns, rested for 30 sec between bursts). Cell debris was removed by centrifugation for 3 min at 5000 g, 4°C, and protein concentration of the resulting samples determined using the Bradford assay. To precipitate protein, samples were mixed with an equal volume of 10% ice-cold trichloroacetic acid (TCA), and precipitated protein removed by centrifugation for 5 min at 5000 g, 4°C. Samples were stored at −70°C until further analysis. To reduce cystine to cysteine, 250 µl sample was mixed with 25 µl 75 mM Tris-(2-carboxyethyl) hydrochloride (TCEP) and incubated for 30 min at RT. Thiol groups were derivatized by incubating 200 µl of the reduced sample with an equal volume of 1 mg/ml 7-fluoro-4-sulfobenofuran (SBD-F, CAS 84806-27-9), 2.5 M boric acid pH 10, for 60 min at 60°C in a shaking water bath. The samples were then cooled on ice, and the supernatant was collected after centrifugation for 5 min at 3000 g. Derivatized samples were separated on a Spherisorb 5u ODS (2), 250×4.6 mm column (Phenomenex) with 0.55 M acetate buffer pH 4.0 mobile phase at a rate of 1 ml/min, on an Ultimate 3000 HPLC instrument with Chromeleon evaluation software (Dionex). The cystine content was determined by fluorimetry (excitation 385 nm, emission 515 nm) by comparison to a standard curve (0.6–12 µM cystine (C_6_H_12_N_2_O_4_S_2_, Sigma C8755) in boric acid buffer [0.1 M H_3_BO_3_, 2 mM EDTA, pH 9.5]), and expressed as µmol cystine/g protein.

## Supporting Information

Figure S1 **HCT116 cells were exposed to different concentrations of b-AP15 for 1 hour, followed by washing and incubation in drug-free medium for 16 hours.** Cell lysates were subjected to immunoblotting for K48-linked ubiquitin, HSP70, HSP90 and β-actin (loading control).
(EPS)

Figure S2 **Cells were treated with or without (control) 10 µM cpdA for 16 hours, exposed to the indicated concentration of b-AP15 for 1 hour, then incubated for a further 16 or 24 hours in drug-free medium.** Following treatment, the number of dead cells was measured by Trypan-blue staining.
(EPS)
